# The Proteoglycan Biglycan Modulates Platelet Adhesion and Thrombus Formation in a GPVI-Dependent Manner

**DOI:** 10.3390/ijms222212168

**Published:** 2021-11-10

**Authors:** Henrike Hoermann, Irena Krueger, Nadine Maurus, Friedrich Reusswig, Yi Sun, Christina Kohlmorgen, Maria Grandoch, Jens W. Fischer, Margitta Elvers

**Affiliations:** 1 Department of Vascular and Endovascular Surgery, Experimental Vascular Medicine, Medical Faculty and University Hospital Düsseldorf, Heinrich-Heine-University Düsseldorf, 40225 Düsseldorf, Germany; Henrike.Hoermann@med.uni-duesseldorf.de (H.H.); Irena.Krueger@med.uni-duesseldorf.de (I.K.); nadine.maurus@web.de (N.M.); FriedrichWilhelmBernd.Reusswig@med.uni-duesseldorf.de (F.R.); 2 Centre of Membrane Proteins and Receptors (COMPARE), Institute of Cardiovascular Sciences, College of Medical and Dental Sciences, University of Birmingham, Birmingham B15 2TT, UK; Y.Sun.3@bham.ac.uk; 3 Institute for Pharmacology und Clinical Pharmacology, University Hospital of the Heinrich-Heine-University, 40225 Düsseldorf, Germany; christina.kohlmorgen@outlook.de (C.K.); maria.grandoch@hhu.de (M.G.); jens.fischer@uni-duesseldorf.de (J.W.F.)

**Keywords:** platelets, biglycan, GPVI, thrombus formation, hemostasis, arterial thrombosis

## Abstract

Background: Vascular injury induces the exposure of subendothelial extracellular matrix (ECM) important to serve as substrate for platelets to adhere to the injured vessel wall to avoid massive blood loss. Different ECM proteins are known to initiate platelet adhesion and activation. In atherosclerotic mice, the small, leucine-rich proteoglycan biglycan is important for the regulation of thrombin activity via heparin cofactor II. However, nothing is known about the role of biglycan for hemostasis and thrombosis under nonatherosclerotic conditions. Methods: The role of biglycan for platelet adhesion and thrombus formation was investigated using a recombinant protein and biglycan knockout mice. Results: The present study identified biglycan as important ECM protein for the adhesion and activation of platelets, and the formation of three-dimensional thrombi under flow conditions. Platelet adhesion to immobilized biglycan induces the reorganization of the platelet cytoskeleton. Mechanistically, biglycan binds and activates the major collagen receptor glycoprotein (GP)VI, because reduced platelet adhesion to recombinant biglycan was observed when GPVI was blocked and enhanced tyrosine phosphorylation in a GPVI-dependent manner was observed when platelets were stimulated with biglycan. In vivo, the deficiency of biglycan resulted in reduced platelet adhesion to the injured carotid artery and prolonged bleeding times. Conclusions: Loss of biglycan in the vessel wall of mice but not in platelets led to reduced platelet adhesion at the injured carotid artery and prolonged bleeding times, suggesting a crucial role for biglycan as ECM protein that binds and activates platelets via GPVI upon vessel injury.

## 1. Introduction

Platelets are small anucleate cells of the hematopoietic system and play a major role in hemostasis. At sites of vascular injury, platelets adhere to the damaged vessel wall to form a hemostatic plug important to avoid blood loss [1]. However, uncontrolled platelet activation can trigger vessel occlusion leading to myocardial infarction or stroke [2]. Injury of the endothelium induces the exposure of the extracellular matrix (ECM) that serves as substrate to initiate the activation, adhesion, and aggregation of circulating platelets [3,4].

The primary physical support for endothelial cells is provided by the vascular endothelial basement membrane and is exposed to circulating platelets following denudation of the endothelium. ECM proteins of the basement membrane are laminin, fibronectin, entactin, several proteoglycans, and collagen type IV [4]. The layers below the basement membrane vary according to the vessel type [3]. The smooth muscle layer between the basement membrane and the internal elastic lamina has been termed vascular ECM and is composed of elastin, microfibrils, collagens, fibronectin, proteoglycans, and vitronectin. The ECM proteins that mediate platelet adhesion and activation vary depending on the type of vessel and the severity of the injury. The major components of the vascular matrix are fibrillar collagens (type I and III) known to capture circulating von Willebrand factor (VWF) to mediate adhesion and activation of platelets through GPVI and integrin α_2_β_1_ [4]. It is well known that major components of the vascular extracellular matrix responsible for the interaction with platelets are laminin, fibronectin, vitronectin, and collagen. Moreover, different structural proteins such as VWF, fibrinogen, and thrombospondin are known to be recruited to the ECM upon injury [3].

The subendothelial ECM regulates platelet adhesion and thrombus formation upon vessel injury. After denudation of endothelial cells, ECM proteins are exposed to the flowing blood, followed by capturing (tethering) of platelets to counter the high shear rates in arteries. Tethering of platelets is mediated by VWF binding to subendothelial collagens via glycoprotein (GP)Ib–IX–V and enables other ECM proteins with slower on-rate to interact with platelet membrane receptors [5]. The interaction of fibrillar collagens with GPVI is a major pathway that induces the activation of platelets, including inside-out activation of integrins to allow stable platelet adhesion at sites of vessel injury via integrin α_IIb_β_3_, α_2_β_1_, α_5_β_1_, and α_6_β_1_ [4,6].

Integrin α_2_β_1_ binds to collagen to induce stable adhesion especially under conditions of reduced VWF plasma concentrations [4]. Moreover, integrin α_2_β_1_ is known to bind to the small proteoglycan decorin to induce platelet adhesion [7]. Biglycan is another small proteoglycan of the ECM and is involved in cell adhesion and migration of fibroblasts and smooth muscle cells in the lung [8,9]. De novo synthesis of biglycan can be triggered in different cell types such as endothelial cells, smooth muscle cells, fibroblasts, and macrophages. Transforming growth factor-beta and platelet-derived growth factor regulate the production of biglycan [10,11]. In macrophages, IL-6 and IL-1β stimulate the synthesis of biglycan [12]. Biglycan is found in the ECM of arterioles and capillaries and is important for thrombin generation upon inflammation and atherosclerosis [13]. Beside its role as a component of the ECM, biglycan may act as a signaling molecule to stimulate proinflammatory signaling such as inflammatory responses to microbial invasion induced by biglycan-mediated clustering of several types of receptors on the cell surface [14]. To this end, biglycan is implicated in kidney disease [15], the regulation of inflammation and autophagy in cancer [16], cardiac hypertrophy [17], erythropoietin production [18], autoimmune perimyocarditis [19], and adaptive remodeling after myocardial infarction [20]. However, the impact of small proteoglycans as ECM proteins of the vessel wall—especially of biglycan—in platelet adhesion and activation in hemostasis and arterial thrombosis is poorly understood. Thus, the present study explored the role and importance of biglycan for platelet adhesion and thrombus formation in vitro and in vivo.

## 2. Results

### 2.1. Minor Expression of Biglycan in Platelets

**Figure 1:**
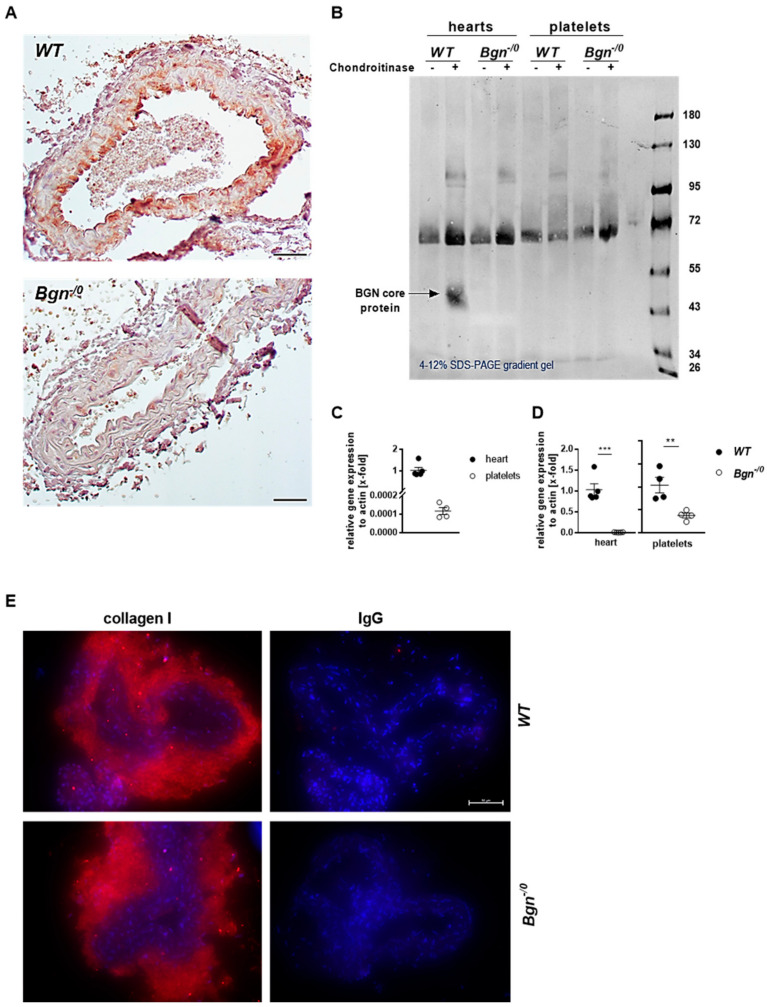
Minor expression of biglycan in platelets. (**A**) Representative images of biglycan in the ECM of the carotid artery. Biglycan was stained in paraffin-embedded sections of the carotid artery of wildtype (upper panel) and *Bgn^-/0^* mice (lower panel) after FeCl_3_-induced injury to confirm the presence of biglycan in the subendothelial layer of the vessel. Biglycan was not present in the vessel wall of biglycan-deficient mice. Nuclei were stained with Mayers hemalum. Scale bar = 100 µm. (**B**) Western blot analysis of biglycan in murine hearts and platelets from wildtype and biglycan-deficient mice. (**C**) Detection of biglycan mRNA in platelets from WT mice using qRT-PCR. mRNA isolated from murine hearts was used as a positive control. Mean values ± SEM, n = 4–5. (**D**) Biglycan expression in platelets and heart tissue from *Bgn^-/0^* mice compared to WT mice using qRT-PCR. Mean values ± SEM, n = 4–5. (**E**) Collagen I distribution (red) in the carotid artery is not altered in biglycan-deficient mice compared to wildtype controls, cell nuclei stained with DAPI (blue), n = 3. Statistical analysis was performed with two-tailed paired Student’s *t*-test, ** *p* < 0.01, *** *p* < 0.001.

Biglycan is a component of the ECM of the vessel wall in myocardial arterioles, of the glycocalyx of capillaries [13], and in the carotid artery after oxidative vessel injury with FeCl_3_ (Figure 1A). As platelets are able to store different ECM proteins in their alpha granules [3], we first analyzed if platelets express biglycan. As shown in Figure 1B, we were not able to detect biglycan in platelets from wildtype (WT) mice using Western blot ana-lysis. In contrast, biglycan was detected in murine hearts that served as positive control. However, quantitative RT-PCR showed little biglycan expression in platelets compared to strong gene expression in heart tissue (Figure 1C,D). Furthermore, we did not observe major alterations in the distribution of collagen I in biglycan-deficient mice compared to controls (Figure 1E).

### 2.2. Platelets Adhere to Immobilized Biglycan and Biglycan-Collagen with Enhanced Surface Coverage

**Figure 2:**
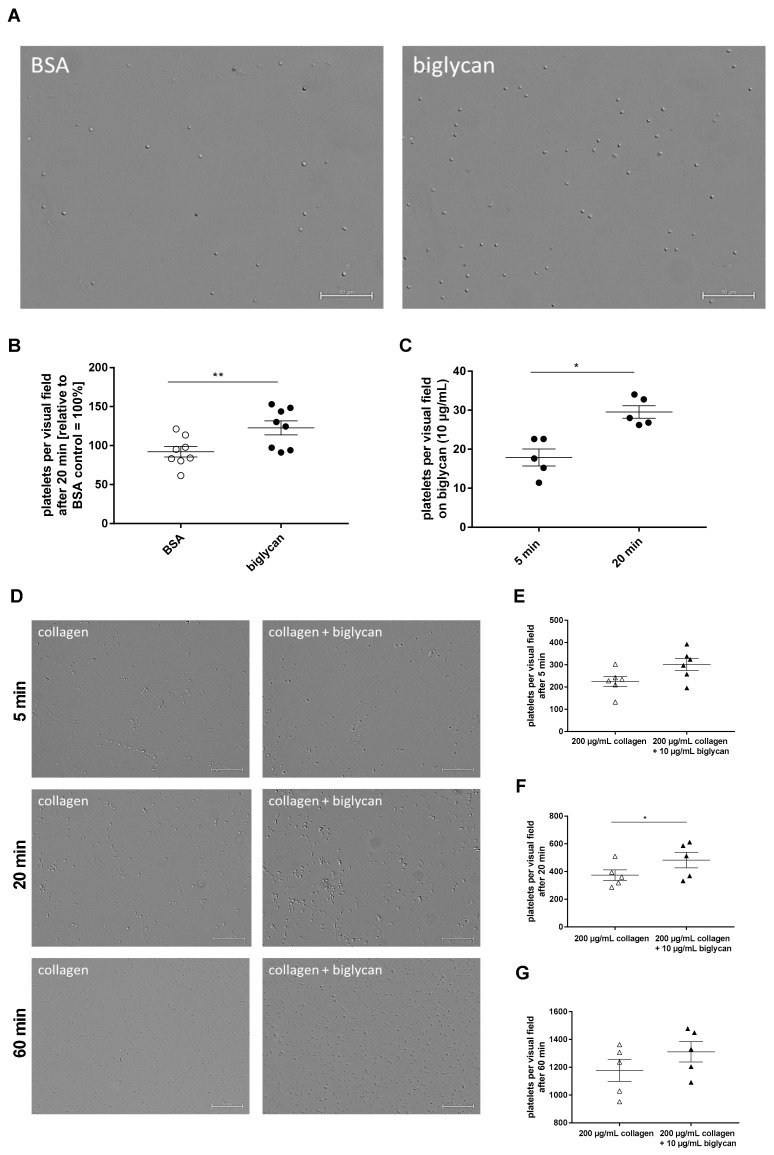
Murine platelets are able to adhere to immobilized biglycan and show enhanced adhesion and surface coverage on a biglycan-collagen matrix. (**A**) Representative images show adherent murine platelets to immobilized recombinant biglycan (10 µg/mL) compared to BSA (10 mg/mL) that serves as negative control, taken 20 min after start of incubation. Scale bar = 50 µm. (**B**) Quantitative analysis of platelet adhesion to biglycan (10 µg/mL) after 20 min compared to adhesion on a BSA matrix (10 µg/mL) = 100%, n = 8. (**C**) Platelet adhesion to immobilized biglycan (10 µg/mL) after 5 and 20 min revealed an elevated number of adherent platelets on biglycan with increased time of incubation, N = 5. (**D**) Representative images show adherent platelets on a collagen matrix (200 µg/mL) compared to a biglycan-collagen (10/200 µg/mL) matrix at different time points. Scale bar = 50 µm. (**E**–**G**) Quantification of adherent platelets after 5 (**E**), 20 (**F**), and 60 min (**G**) of incubation on collagen (200 µg/mL) and biglycan (10 µg/mL)-collagen (200 µg/mL) matrices. Mean values ± SEM. Statistical analysis was performed with two-tailed paired Student’s *t*-test, * *p* ≤ 0.05, ** *p* < 0.01.

**Figure 3:**
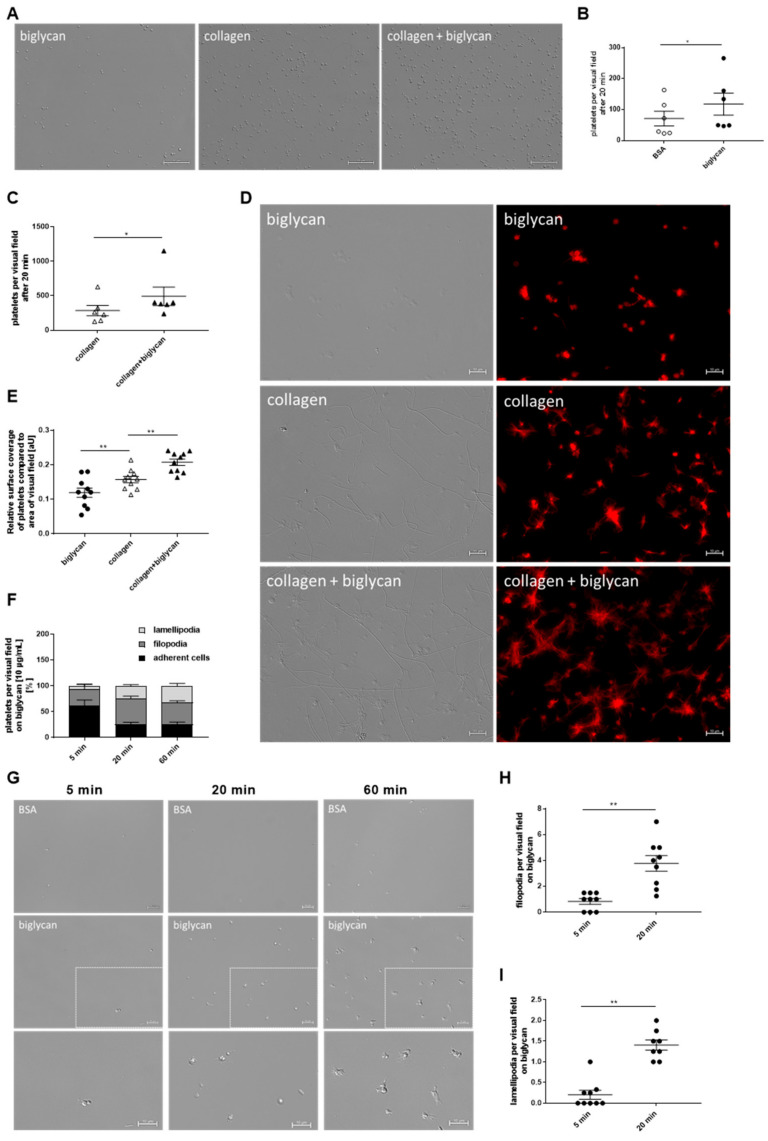
Adhesion and spreading of human platelets on immobilized biglycan. (**A**–**C**) Adhesion of human platelets on immobilized biglycan, collagen, and biglycan-collagen. (**A**) Representative images show adherent platelets on a recombinant biglycan (10 µg/mL), collagen (200 µg/mL), and biglycan-collagen (10/200 µg/mL) matrix, scale bar = 50 µm. (**B**) Enhanced adhesion of human platelets on immobilized biglycan (10 µg/mL) compared to BSA (10 mg/mL) control matrix after 20 min, n = 6, mean values ± SEM. Statistical analysis was performed with two-tailed, paired Student’s *t*-test. (**C**) Enhanced adhesion of human platelets on a biglycan-collagen (10/200 µg/mL) matrix compared to collagen (200 mg/mL) after 20 min, n = 6, mean values ± SEM. Statistical analysis was performed with two-tailed Wilcoxon matched-pairs signed-rank test. (**D**) F-actin staining of human platelets that were allowed to spread on biglycan (10 µg/mL), collagen (200 µg/mL) and biglycan-collagen (10/200 µg/mL) for 20 min. Representative pictures of differential interference contrast (DIC, left panel) and fluorescence microscopy (right panel), scale bar = 10 µm, and (**E**) quantification of the surface coverage of spread platelets on a biglycan-collagen matrix compared to biglycan or collagen alone in relation to the total amount of platelets per visual field using ImageJ (Wayne Rasband), n = 10, mean values ± SEM, statistical analysis using one-way ANOVA followed by Sidak’s post hoc test. (**F**–**I**) Spreading of human platelets on immobilized recombinant biglycan using differential interference contrast (DIC) microscopy. (**F**) Quantification of platelets that adhere or form filopodia or lamellipodia on immobilized biglycan (10 µg/mL) at indicated time points in percent of all platelet per visual field, n = 9, mean values ± SEM. Statistical analysis using two-way ANOVA followed by Tukey’s post hoc test. (**G**) Representative images of platelet spreading on a BSA (10 mg/mL) control matrix (upper panel) and recombinant biglycan (10 µg/mL) (middle panel). A picture detail of platelets on recombinant biglycan (middle panel) is shown in the lower panel, scale bar = 10 µm. Increased filopodia (**H**) and lamellipodia (**I**) formation of human platelets on recombinant biglycan (10 µg/mL) after 20 min was observed. Data represent mean values ± SEM, n = 8–9, statistical analysis was performed using two-tailed Wilcoxon matched-pairs signed-rank test. * *p* < 0.05, ** *p <* 0.01.

To investigate if platelets are able to adhere to biglycan, purified recombinant biglycan was immobilized onto coverslips and murine platelets were allowed to adhere to the biglycan matrix. After 5, 20, and 60 min, significantly enhanced platelet adhesion to immobilized biglycan was observed compared to the BSA control (Figure 2A,B, Supplementary Figure S1A–C). Furthermore, enhanced platelet adhesion to immobilized biglycan was observed when we compared platelet adhesion to biglycan after 5 and 20 min (Figure 2C). We next compared platelet adhesion to a collagen (native equine tendon type I collagen) and a biglycan-collagen matrix because biglycan is known to directly interact with fibrillar type I collagen to modulate fibril formation, growth, and morphology [21]. As shown in Figure 2D,F, platelet adhesion to a biglycan-collagen matrix was significantly enhanced after 20 min compared to collagen alone. However, no differences in platelet adhesion were observed at early and late time points (5 and 60 min) (Figure 2D,E,G). We also investigated the adhesion of human platelets to immobilized biglycan compared to a collagen, a biglycan-collagen, and a BSA control matrix (Figure 3A–C; Supplementary Figure S1D,E). Again, increased platelet adhesion on a biglycan-collagen matrix compared to on collagen alone was observed after 20 min (Figure 3C).

### 2.3. Platelet Adhesion to Immobilized Recombinant Biglycan Induced Cytoskeletal Reorganization and Elevated Integrin Activation in the Presence of Biglycan

Next, we performed F-actin staining of the cytoskeleton of spread platelets with Alexa-Phalloidin 568, which revealed increased surface coverage of platelets on a biglycan-collagen matrix compared to on biglycan or collagen alone (Figure 3D,E). Moreover, we examined biglycan-induced reorganization of the platelet cytoskeleton in spreading experiments by the analysis of filopodia and lamellipodia formation using differential interference contrast (DIC) microscopy. Human platelets were allowed to adhere to immobilized biglycan for 5, 20, and 60 min, and the numbers of platelets that adhere to form filopodia and lamellipodia were counted, respectively (Figure 3F–I). After 5 min of platelet adhesion to immobilized biglycan, only 7.6% of platelets were able to form lamellipodia. The number of platelets with lamellipodia increased over time, reaching 23.02% (after 20 min) and 31.58% (after 60 min) of all platelets counted on the biglycan matrix. Filopodia formation was also enhanced over time, showing that 33.49% of platelets were able to induce filopodia formation on immobilized biglycan after 5 min, while 51.32% (after 20 min) and 41.45% (after 60 min) of platelets showed filopodia formation at later time points.

**Figure 4:**
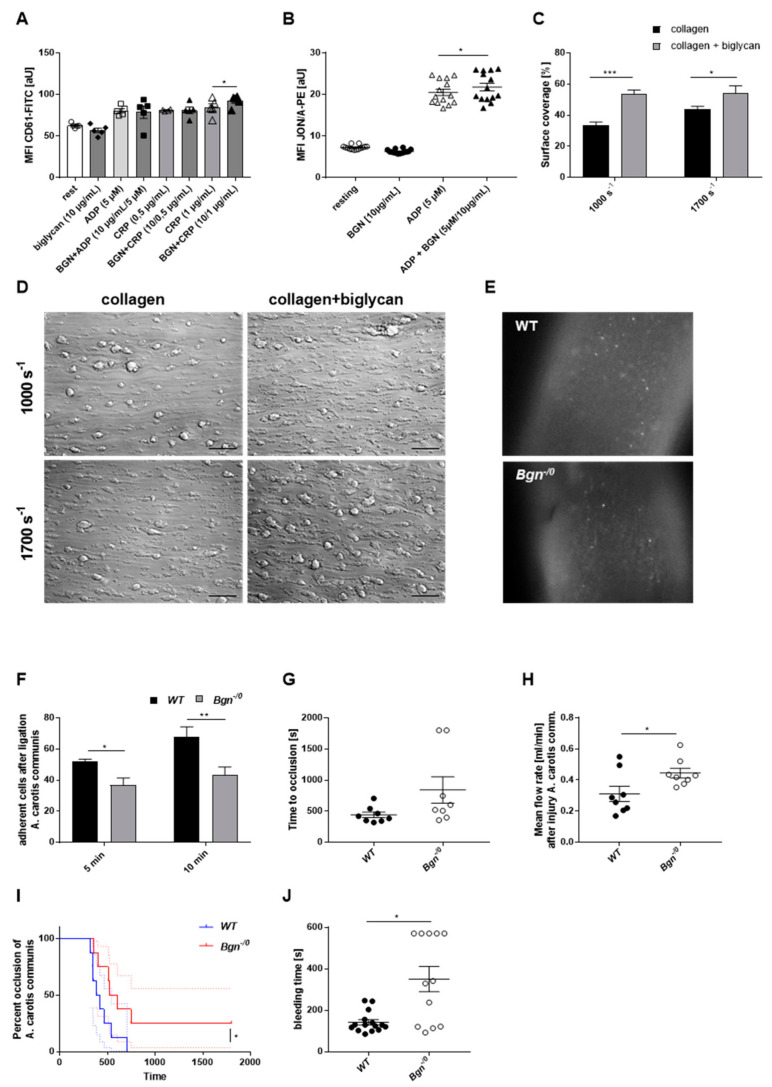
The presence of biglycan induced elevated murine platelet activation and thrombus formation ex vivo and in vivo. (**A**) Increased exposure of integrin α_IIb_β_3_ (CD61) after platelet stimulation using collagen-related peptide (CRP) with and without recombinant biglycan and (**B**) increased activation of integrin α_IIb_β_3_ (MFI of JON/A) following platelet stimulation using ADP with and without recombinant biglycan as measured by flow cytometric analysis of wildtype platelets. Mean fluorescence intensity (MFI) was determined, n = 4–5 (**A**), n = 14 (**B**), mean values ± SEM are shown. Statistical analysis was performed using one-way ANOVA followed by Sidak’s post hoc test (A) and two-tailed Wilcoxon matched-pairs signed-rank test (**B**). (**C**,**D**) Whole blood from *C57BL/6J* mice was perfused through a flow chamber system. (**C**) Increased thrombus formation on a biglycan-collagen (10/200 µg/mL) matrix compared to on collagen (200 µg/mL) alone at shear rates of 1000 s^−1^ and 1700 s^−1^, n = 5–8, bar graphs depict mean values ± SEM, statistical analysis was performed with two-way ANOVA followed by Sidak’s post hoc test. (**D**) Representative images of thrombus formation on a collagen and a biglycan-collagen matrix, scale bar = 50 µm. (**E**,**F**) Using in vivo video microscopy, fluorescently labeled platelets adherent to the injured carotid artery were detected and quantified. *Bgn^-/0^* mice showed reduced platelet adhesion to the injured vessel wall 5 min and 10 min after ligation compared to wildtype littermates (WT). (**E**) Representative images of adherent platelets at the injured vessel wall of the carotid artery in *Bgn^-/0^* and WT mice that received fluorescently labelled platelets of their own genotype are shown. (F) Quantification of the number of adherent platelets, n = 4–5, bar graphs depict mean values ± SEM, statistical analysis was performed with two-way ANOVA followed by Sidak’s post hoc test. (**G**–**I**) Injury of the carotid artery with FeCl_3_ and determination of the time to occlusion (**G**), analysis of the mean flow rate (**H**), and the percentage of vessel occlusion in *Bgn^-/0^* and WT mice (**I**), n = 8, statistical analysis was performed with two-tailed, Mann–Whitney test (**G**), two-tailed, unpaired Student’s *t*-test (**H**) and log-rank (Mantel–Cox) test (**I**). (**J**) The bleeding time as marker for hemostasis was determined in *Bgn^-/0^* and WT mice. Time to cessation of bleeding was measured; in case of no cessation of bleeding, measurements had to be stopped after 570 s due to high blood loss. Mean values ± SEM are shown, n = 12–15, Statistical analysis was performed with two-tailed Mann–Whitney test. * p < 0.05, ** p < 0.01, *** p < 0.001.

Subsequently, we explored if the presence of biglycan has any impact on platelet activation. Therefore, the exposure and activation of integrin α_IIb_β_3_ were determined by flow cytometry. Biglycan alone neither up-regulates integrin α_IIb_β_3_ at the platelet surface nor induces integrin activation. However, in the presence of biglycan, the stimulation of the major collagen receptor GPVI with low dose of CRP (1 µg/mL), compared to CRP alone, led to an enhanced exposure of integrin α_IIb_β_3_ (Figure 4A). Furthermore, integrin activation, but not the expression of integrin α_IIb_β_3_ at the platelet surface, was increased by co-stimulation of platelets with low dose of ADP and with biglycan compared to with ADP alone (Figure 4B). In contrast, determination of P-selectin at the platelet surface revealed no differences when platelets were co-stimulated with biglycan (data not shown).

### 2.4. Enhanced Thrombus Formation under Flow Conditions in the Presence of Recombinant Biglycan

To investigate the relevance of biglycan under more physiological conditions, we analyzed platelet adhesion and the formation of three-dimensional thrombi under flow conditions. Whole blood was perfused over a collagen-coated and a collagen-biglycan-coated surface at arterial shear rates (1000 s^−1^ and 1700 s^−1^), and thrombus formation was analyzed (Figure 4C,D). Determination of the surface covered by thrombi revealed enhanced thrombus formation on a biglycan-collagen matrix compared to on collagen alone at both shear rates tested.

We detected biglycan expression in platelets (Figure 1). Therefore, we investigated if biglycan deficiency impacts platelet adhesion and thrombus formation on a collagen-coated surface under flow. To this end, we first characterized blood cell counts and mean platelet volume (MPV) of wildtype and biglycan-deficient mice and found no alterations between the groups (Supplementary Table SI). Furthermore, glycoprotein expression at the platelet surface and platelet activation as determined by P-selectin exposure and activated integrin α_IIb_β_3_ were not altered following platelet stimulation with different agonists (Supplementary Figure S2A–C). Flow chamber experiments revealed unaltered thrombus formation under shear rates of 1000 s^−1^ and 1700 s^−1^ (Supplementary Figure S2D) when we used whole blood from *Bgn^-/0^* mice compared to wildtype controls. Thrombus formation was also investigated in a second ex vivo model where the occlusion of a capillary was analyzed with the Total Thrombus-formation Analysis System (T-TAS^®^). At shear rates of 600 s^−1^, no differences were observed when the occlusion of the capillary was measured after perfusion of whole blood from biglycan-deficient and control mice. Full occlusion of the collagen-coated capillary was achieved within 374.8 ± 49.2 s (wildtype) and 310 ± 10.82 s (*Bgn^-/0^*) (Supplementary Figure S2E,F). Moreover, we analyzed thrombin plasma levels using whole blood from wildtype and biglycan-deficient mice (Supplementary Figure S3A–E) as it is known that the loss of biglycan in apolipoprotein (Apo) E-deficient mice leads to enhanced platelet activation accompanied by enhanced thrombin generation [13]. Unaltered platelet activation (Supplementary Figure S2B,C) was in line with unaltered thrombin plasma levels in biglycan-deficient mice (Supplementary Figure S3A–E) although the time to peak was enhanced using biglycan-deficient platelets (Supplementary Figure S3D). Accordingly, clot retraction using PRP from biglycan-deficient and wildtype mice was unaltered as well (Supplementary Figure S3F,G).

### 2.5. Reduced Platelet Adhesion to the Injured Carotid Artery and Prolonged Bleeding Times in Mice Lacking Biglycan

Our results signify that biglycan affects the reorganization of the platelet cytoskeleton, platelet adhesion and activation, and thrombus formation. Furthermore, soluble and immobilized biglycan were shown to support platelet adhesion and thrombus formation in the presence of GPVI agonists. To investigate the role of biglycan in shear-dependent platelet adhesion in vivo, we determined the number of adherent platelets to the injured carotid artery at different time points after ligation with a surgical thread for 5 min (Figure 4E,F). Wildtype mice received fluorescently labeled platelets from wildtype controls and biglycan-deficient mice received platelets from biglycan-deficient mice. Using in vivo video microscopy, we were able to determine the number of fluorescent platelets adhering to the damaged vessel wall 5 min after ligation of the carotid artery; a significant reduction of adherent platelets in biglycan-deficient (36.69 ± 3.86 platelets/mm^2^) compared to control mice (51.81 ± 1.41 platelets/mm^2^) was observed (Figure 4E,F). A significant reduction of adherent platelets was also observed after 10 min following ligation of the carotid artery of biglycan-deficient (43.48 ± 4.45 platelets/mm^2^) compared to control mice (67.94 ± 5.50 platelets/mm^2^). Next, we analyzed the occlusion of the carotid artery after FeCl_3_-induced injury of the vessel (Figure 4G–I). Significant differences between biglycan-deficient and wildtype mice were observed when we determined the mean flow rate after vessel injury and the percentage of occlusion of the carotid artery (Figure 4H,I). However, no alterations were detected when we determined the time to occlusion of the vessel after FeCl_3_-induced injury of wildtype and biglycan-deficient mice (Figure 4G).

To analyze the role of biglycan for hemostasis, tail bleeding times of biglycan-deficient and wildtype controls were performed and the tail tip of mice was amputated (Figure 4J). Time to arrest bleeding at sites of a defined tail wound was enhanced in biglycan-deficient compared to control mice (Figure 4J). To investigate if defective hemostasis is caused by biglycan deficiency in the ECM of the vessel wall or by biglycan-deficient platelets, we determined tail bleeding times in bone marrow chimeric mice. Before we started the experiments, the number of platelets was measured in bone marrow chimeric mice 6 weeks after transplantation (Supplementary Figure S4A). Again, biglycan-deficient mice that received bone marrow from wildtype controls showed enhanced bleeding times compared to wildtype mice that received bone marrow from biglycan-deficient mice, suggesting that deficiency of biglycan in the ECM of the vessel wall is responsible for altered hemostasis (Supplementary Figure S4B). Again, no differences were observed when the time to occlusion was determined after FeCl_3_-induced injury of the carotid artery using bone marrow chimeric mice (Supplementary Figure S4C). However, a decreased percentage of vessel occlusion of Bgn-deficient mice that received bone marrow from wildtype controls was determined by trend (Supplementary Figure S4D).

### 2.6. Platelets Adhere to Biglycan via the Collagen Receptor GPVI

**Figure 5:**
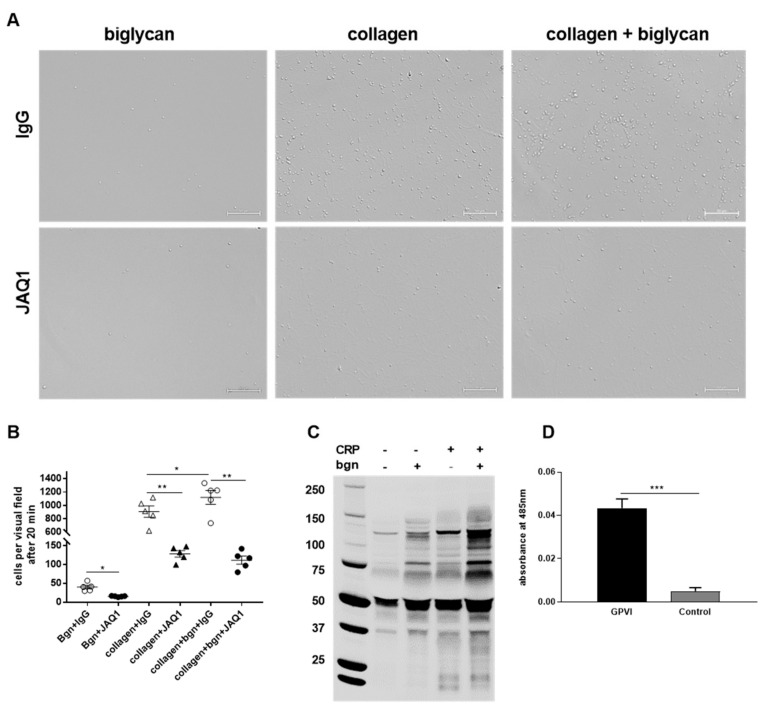
Impact of GPVI on platelet adhesion to biglycan. (**A**,**B**) Reduced platelet adhesion to immobilized biglycan (10 µg/mL), collagen (200 µg/mL), and biglycan-collagen (10/200 µg/mL) after blocking of GPVI by antibody treatment (JAQ1, Emfret analytics, 2 µg/mL). Control platelets were treated with an IgG antibody. (**A**) Representative images and (**B**) quantitative analysis of adherent platelets. Scale bar = 50 µm, n = 5, mean values ± SEM. Statistical analysis was performed with one-way ANOVA followed by Sidak’s post hoc test. (**C**) Western blots show protein tyrosine phosphorylation of platelets stimulated with collagen-related peptide (CRP) and recombinant biglycan. Enhanced phosphorylation was detected in platelets stimulated with CRP (5 µg/mL) and biglycan (10 µg/mL) compared to with CRP or biglycan alone, n = 5. (**D**) Pentameric GPVI binds to BGN (100 ng/uL immobilized on MaxiSorp plate), while a negative control protein (ACVR1) does not, n = 3–5, two-tailed, unpaired Student’s *t*-test with Welch’s correction. * *p* < 0.05, ** *p* < 0.01, *** *p* < 0.001.

To identify a role for GPVI in platelet adhesion to a biglycan and a biglycan-collagen surface, platelets were allowed to adhere to different matrices in the presence and absence of the GPVI-blocking antibody JAQ1 (Emfret analytics). As shown in Figure 5A,B, platelet adhesion to collagen (used as control), to immobilized biglycan, and to a biglycan-collagen surface was significantly reduced when GPVI was blocked by antibody treatment, suggesting that GPVI is involved in platelet binding/adhesion to biglycan. To investigate the impact of GPVI in platelet adhesion to biglycan in further detail, we stimulated platelets with biglycan and analyzed if biglycan is able to modify the phosphorylation of GPVI target proteins. As shown in Figure 5C, biglycan is able to stimulate tyrosine phosphorylation in a GPVI-dependent manner. Furthermore, enhanced tyrosine phosphorylation was observed by simultaneous activation of platelets with CRP and biglycan compared to with CRP alone (Figure 5C). Importantly, direct binding of pentameric GPVI to BGN, but no binding with control protein (ACVR1), was observed using the microarray AVEXIS screening assay (Figure 5D). Taken together, our data strongly imply that platelet binding to biglycan and biglycan/collagen is mediated by GPVI.

## 3. Discussion

In this study, we have shown that genetic deletion of the small proteoglycan biglycan interferes with hemostasis and protects against arterial thrombosis induced by reduced platelet binding to the injured vessel. Mechanistically, platelets are capable to bind to immobilized and soluble biglycan to increase GPVI signaling via tyrosine phosphorylation, leading to platelet degranulation and β_3_-integrin activation important for platelet adhesion and thrombus formation. Therefore, the current findings add important new information about the role of small proteoglycans, especially of biglycan, in arterial thrombosis and hemostasis.

The impact of biglycan in cardiovascular diseases was already shown earlier. Loss of biglycan led to enhanced thrombin generation and platelet activation in ApoE-deficient mice, suggesting a role for biglycan in inflammation and atherosclerosis [13]. In contrast, no major alterations were observed in thrombin plasma levels in biglycan-deficient mice on a C57BL/6 genetic background (Supplementary Figure S3A–E), suggesting that the role of biglycan as regulator of thrombin activity by heparin cofactor II is more pronounced in proinflammatory, atherosclerotic mice compared to healthy mice. Accordingly, platelet activation was unaltered in biglycan-deficient mice compared to control mice (Supplementary Figure S2B,C). In contrast to enhanced platelet adhesion at the uninjured carotid artery in ApoE/biglycan double-deficient mice, reduced platelet adhesion was measured at the ligated carotid artery in *Bgn^-^*^/0^ mice in the present study. Therefore, it is apparent that biglycan has different roles in regulating platelet function in hypercholesterinemic ApoE-deficient mice compared to wildtype controls. Possible mechanisms may include the presence of an intact glycocalyx in the uninjured carotid artery of ApoE-deficient mice versus the destruction of the glycocalyx and exposure of the subendothelial matrix in the present model. In summary, both studies revealed that the function of biglycan in the glycocalyx varies compared to its functions in the arterial wall.

After myocardial infarction, biglycan plays a major role for stable collagen matrix formation and for the preservation of cardiac left ventricle function [20]. In addition, an involvement of biglycan in aortic dissection of mice has been described [22]. Different studies provided evidence that biglycan exerts proinflammatory effects via toll-like receptor (TLR)2 and TLR4, suggesting a role in pathogen-mediated and sterile inflammation [12,14,23,24,25]. Here we provided evidence for a role of biglycan in platelet adhesion. So far, only one study exists that has analyzed the role of proteoglycans in platelet adhesion and activation. In specific, the small proteoglycan decorin was shown to support platelet adhesion and activation. Unfortunately, the authors did not analyze the impact of decorin in platelet adhesion and thrombus formation in arterial thrombosis and hemostasis in vivo [7]. Interestingly, decorin was shown to bind to the collagen receptor α_2_β_1_ integrin to induce tyrosine phosphorylation. Here, we provided evidence for biglycan-mediated platelet adhesion and activation via the collagen receptor GPVI, because blocking of the receptor by antibody treatment reduced platelet adhesion to biglycan, collagen, and a mixed biglycan-collagen matrix. As biglycan is described to bind and stabilize the collagen fibrils in the ECM of the vessels [21] it can be feasible that biglycan functions as a co-factor for collagen binding to GPVI, thus supporting an enhancement in platelet adhesion, integrin activation, and thrombus formation. However, we observed significant differences in platelet adhesion to biglycan alone when we blocked GPVI with an antibody (JAQ1, Figure 5B). This supports the hypothesis that biglycan is able to induce signaling via GPVI in the absence of collagen.

Furthermore, we detected tyrosine phosphorylation of biglycan in a GPVI-dependent manner. In contrast to Guidetti and colleagues, we found a role for biglycan in arterial thrombosis that is causative for myocardial infarction and stroke. Decreased platelet adhesion of biglycan-deficient platelets to the injured carotid artery was observed at early and intermediate time points (5 and 10 min), while we were not able to detect any differences compared to control mice after 30 min (data not shown), suggesting that the defects induced by the loss of biglycan can be compensated over time. Strikingly, defects in hemostasis were observed in individual biglycan-deficient mice, while others were able to compensate the loss of biglycan and showed normal bleeding times compared to control mice. Importantly, measurements of bleeding time had to be terminated in biglycan-deficient mice with defective hemostasis after 570 s because of elevated blood loss, suggesting that mice, which cannot compensate for the loss of biglycan, exert strong platelet activation defects.

In the last years, new GPVI ligands have been identified, including diesel exhaust particles (DEP) and large polysaccharides such as fucoidan and dextran sulfate [26]. Recently, fibrin has been identified as ligand for monomeric GPVI [27]. For the first time, we provide evidence for biglycan as novel ligand for GPVI in the presented study. First, tyrosine phosphorylation in a GPVI-dependent manner was detected when we stimulated platelets with soluble biglycan. Second, antibody-mediated inhibition of GPVI reduced the adhesion of platelets to immobilized biglycan, suggesting that biglycan can exert its effects via GPVI in an immobilized as well as in a soluble conformation. Direct binding of biglycan and GPVI was initially assumed when we measured reduced platelet adhesion to a pure biglycan matrix in the presence of GPVI-blocking antibodies but in the absence of collagen, which suggests a direct and collagen-independent interaction of biglycan and GPVI. Indeed, Microarray AVEXIS screening confirmed direct binding of biglycan and GPVI.

However, it is not clear if biglycan-induced effects on GPVI activation are mediated via GPVI clustering as shown for platelet adhesion to collagen that induced GPVI dimer clustering. In principle, biglycan is able to cluster several types of receptors and orchestrate their signaling. In a multiple receptor crosstalk, biglycan induces clustering of TRL2/4 and P2X7, which leads to proinflammatory signaling via TNF-α and IL-1β [25]. Thus, it is tempting to speculate that biglycan exerts GPVI signaling via GPVI clustering. Interestingly, fibrin binds selectively to monomeric GPVI, in contrast to collagen, which binds primarily to dimeric GPVI [28,29]. Thus, further analysis of the mechanism of how biglycan affects GPVI signaling is important to validate if specific blocking of biglycan binding to GPVI might be a new antithrombotic target in future.

Beside its role in cardiovascular diseases, biglycan is important for the mineralization of bones [30] and is highly expressed in cancer with metastatic activity and lower survival [31]. Upon tissue stress or injury, the sequestered form of biglycan—that cannot act as a signaling molecule [12]—becomes proteolytically released from the matrix to signal to the immune system [32]. Binding of soluble biglycan to TLR2/4 mediates the activation of the NLRP3 inflammasome [25], suggesting a prominent role for biglycan in different inflammatory diseases. Since platelets express TLR4 and bind to neutrophils in the capillaries of lung and liver upon sepsis [33], it is tempting to speculate that soluble biglycan is able to bind to platelet TLR4 to mediate platelet-induced inflammatory responses. Furthermore, biglycan-mediated activation of the NLRP3 inflammasome includes the formation of reactive oxygen species (ROS) [34,35]. Interestingly, GPVI activation is also involved in ROS generation [36], suggesting that biglycan might be able to induce ROS production of platelets via GPVI. Whether biglycan is also involved in platelet-mediated inflammation and/or ROS generation must be investigated in the future.

## 4. Materials and Methods

### 4.1. Animals

Gene-targeted mice lacking biglycan (*Bgn^-/0^*) and the corresponding wildtype (WT) littermates were bred from breeder pairs and genotyped by PCR. Experiments were performed with male mice aged 2–4 months. C57BL/6J mice (Janvier Labs, Le Genest-Saint-Isle, France) were used for blood withdrawal and organ removal. All animal experiments were conducted according to the Declaration of Helsinki and German law for the welfare of animals. The protocol was approved by the Heinrich-Heine-University Animal Care Committee and by the district government of North Rhine-Westphalia. Permit numbers: 84-02.04.2014.A303; 84-02.05.20.12.284; 84-02.04.2013.A210; 84-02.05.40.16.073.

### 4.2. Murine Platelet Preparation

Platelets were prepared as previously described [37,38]. Murine blood was withdrawn from the retro-orbital plexus under anesthesia and collected in 300 μL heparin (20 U/mL) (Ratiopharm GmbH, Ulm, Germany, # 002304). The blood was centrifuged at 250× *g* for 5 min at 22 °C. The gained supernatant was centrifuged at 50× *g* for 6 min to obtain platelet-rich plasma (PRP). PRP was washed twice at 650 g for 5 min. The remaining pellet was resuspended in murine Tyrode’s buffer (136 mM NaCl, 0.4 mM Na_2_HPO_4_, 2.7 mM KCl, 12 mM NaHCO_3_, 0.1% glucose, 0.35% bovine serum albumin (BSA), pH 7.4), apyrase (0.02 U/mL) (Grade III, from potatoes, Sigma-Aldrich, Burlington, MA, USA, A7646), and prostacyclin (0.5 µM) (Calbiochem, San Diego, CA, USA) and centrifuged at 650× *g* for 5 min. Depending on the following experiment, platelets were either resuspended in Tyrode’s buffer or Tyrode’s buffer plus added CaCl_2_ (0.1 M).

### 4.3. Human Platelet Preparation

Blood samples from healthy volunteers were collected with the approval of the ethics committee of the Heinrich-Heine-University. Participants provided informed consent to their participation in the study (patient’s consent): Permit/Study Number 4669, ID No. 2014042327.

Platelets were prepared as previously described [39]. Fresh citrate-anticoagulated blood was collected from healthy blood donors. The blood samples were centrifuged at 200× *g* for 10 min. The PRP was separated, and phosphate-buffered saline (PBS) (pH 6.5, D8537), 2.5 U/mL apyrase Grade III from potatoes (Sigma-Aldrich, Burlington, MA, USA), and 1 μM prostacyclin (Calbiochem, San Diego, CA, USA, # 538925) were added in a 1:1 volumetric ratio and centrifuged at 1000× *g* for 6 min. The platelet pellet was resuspended in human Tyrode’s buffer (137 mM NaCl, 2.8 mM KCl, 12 mM NaHCO_3_, 0.4 mM Na_2_HPO_4_, 5.5 mM Glucose, 0.1% HIBSA, pH 7.4).

### 4.4. Microarray AVEXIS Screening

Printed slides were rinsed three times with MilliQ H_2_O and blocked with PBS (pH 6.5, # D8537) containing 1% BSA and 10 mM D-biotin for 45 min before interaction screening. Slides were incubated with normalized prey proteins for 1 h, washed three times in PBS/0.5% Tween, and incubated with 1:1000 anti-Flag horseradish peroxidase antibody (Sigma-Aldrich, Burlington, MA, USA) for 1 h. Detection was performed with TSA Alexa 555 substrate (Thermo Fisher Scientific Inc., Waltham, MA, USA) for 1 h. Slides were washed three times in PBS containing 0.1% Tween 20 with gentle rocking between all incubation steps. All steps were performed under condition of 22 °C. Identification of positive interactions was done by scanning slides with a ScanArray Express Microarray Scanner (PerkinElmer, Waltham, MA, USA) at a 550 nm wavelength.

### 4.5. Flow Cytometry

An amount of 100 μL murine blood was collected in 300 μL heparin (20 U/mL), then 500 μL murine Tyrode’s buffer was added and samples were centrifuged at 650× g for 5 min. The remaining pellet was resuspended in 500 μL Tyrode’s buffer and washed again two times as described above. Afterwards, the remaining pellet was resuspended in 500 μL Tyrode’s buffer supplemented with CaCl_2_ (0.1 M). Platelets were activated with one of the following agonists: collagen-related peptide (CRP)-XL (University of Cambridge, United Kingdom), Adenosine 5’-diphosphate (ADP) (Sigma-Aldrich, Burlington, MA, USA, # 01905), U46619 (Tocris Bioscience, Bristol, United Kingdom, # 1932), recombinant human biglycan (R&D Systems, Minneapolis, MI, USA, # 2667-CM-050), protease-activated receptor 4 (PAR-4) peptide (JPT Peptide Technologies GmbH, Berlin, Germany # 16035374). FITC-labeled rat anti-mouse P-selectin (JON/A-PE) monoclonal antibody (M130-1), PE-labeled rat anti-mouse integrin α_IIb_β_3_, monoclonal antibody (M023-2), and FITC-labeled CD61 antibody (M031-1) (all purchased from Emfret Analytics, Würzburg, Germany) were used for labeling. The mean fluorescence intensity (MFI) was measured by flow cytometry.

### 4.6. Platelet Adhesion and Spreading on Immobilized Recombinant Biglycan and Collagen

Experiments were performed as previously described [37,40]. Cover slips were coated with 200 μg/mL type I collagen (Takeda GmbH, Konstanz, Germany), 10 μg/mL recombinant human biglycan (R&D Systems, Minneapolis, MI, USA, # 2667-CM-050), 200 μg/mL type I collagen plus 10 μg/mL recombinant biglycan, or BSA (Sigma-Aldrich, Burlington, MA, USA, # A7906) at a defined area (10 × 10 mm) and incubated at 4 °C overnight. Afterwards, they were blocked with 1% BSA for 60 min. Isolated platelets (8 × 10^4^) (see Section 4.2) were resuspended in 70 μL Tyrode’s buffer supplemented with CaCl_2_ (0.1 M), applicated on the prepared cover slips, and incubated at room temperature for indicated time points. Non-adherent platelets were carefully removed by rinsing with PBS (pH 6.5, D8537). The adherent platelets were fixed with phosphate-buffered formaldehyde (Roti^®^-Histofix 4%, Carl Roth GmbH + Co. KG, Karlsruhe Germany), covered using Fluoromount™ (Sigma-Aldrich, Burlington, MA, USA, # F4680), and imaged using Microscope Axio Observer.D1 (Carl Zeiss Microscopy GmbH, Oberkochem, Germany, AxioCam MRm, objective LD Plan-Neofluar 40× 0.6 Korr. Ph2 M27) for 40 × magnification. The adherent cells were counted with ImageJ software. In case of the static adhesion assays with the GPVI inhibitor JAQ1, platelets were preincubated with 2 μg/mL JAQ1 (Emfret Analytics, Würzburg, Germany, # M011-0) and IgG control for 5 min prior to the application on the cover slips.

For spreading analysis of isolated human platelets, the adherent platelets were rinsed, fixed, and covered as described above. The samples were imaged using Microscope Axio Observer.D1 (Carl Zeiss Microscopy GmbH, Oberkochem, Germany, AxioCam MRm, objective Plan Apochromat 100×/1.40 Oil DIC M27) for differential interference contrast imaging. Platelets that show only filopodia were counted as filopodia-forming cells, platelets that start to form lamellipodia and fully spread platelets were counted as lamellipodia-forming platelets, and landing and attached platelets were counted as adherent cells according to Aslan and McCarty, 2012 [41] using Carl Zeiss Software “ZEN 2012“ (blue edition).

For F-actin staining, 4 × 10^4^ platelets were allowed to adhere on the above-described matrices for 20 min, rinsed with PBS, and fixed with PHEM buffer (100 mM PIPES, 5.25 mM HEPES, 10 mM EGTA, 20 mM MgCl_2_, pH 6.8), blocked with BSA (5%), washed with PBS (pH 6.5, D8537), and incubated with Alexa Phalloidin 568 (Thermo Fisher Scientific Inc., Waltham, MA, USA, # R415) overnight at 4 °C. The samples were rinsed and mounted using Fluoromount™ (Sigma-Aldrich, Burlington, MA, USA, # F4680), and visualized with the Microscope Axio Observer.D1 (Carl Zeiss Microscopy GmbH, Oberkochem, Germany, AxioCam MRm, objective Plan Apochromat 100×/1.40 Oil DIC M27) in differential interference contrast and Cy3 fluorescence microscopy (excitation wavelength 550/emission wavelength 570) channels. In this experiment, the surface coverage of the adherent platelets in relation to the total amount of platelets per visual field was analyzed using ImageJ software (ImageJ-win64).

### 4.7. Thrombus Formation in a Parallel-Plate Flow Chamber System

Thrombus formation under flow was analyzed using a parallel-plate flow chamber system as previously described [39]. Cover slips were coated with 200 μg/mL type I collagen (Collagen Reagens HORM^®^, Takeda GmbH, Konstanz, Germany) or 10 μg/mL recombinant human biglycan (R&D Systems, Minneapolis, MI, USA, # 2667-CM-050) and 200 μg/mL type I collagen at a defined area at 4 °C overnight. The coated surfaces were blocked with 1% BSA (Sigma-Aldrich, Burlington, MA, USA) for 60 min, and the cover slips were installed in the parallel-plate flow chamber system. Whole heparinized (20 U/mL) murine blood was pooled from mice with the same genotype to reach the requested amount of 0.9 mL. The blood was perfused through the flow chamber with continuous flow and different shear rates (1000 s^−1^ and 1700 s^−1^). After 3 min of flow, 5 pictures were taken from different representative areas (400× total magnification, Microscope Axio Observer.D1 (Carl Zeiss Microscopy GmbH, Oberkochem, Germany). The total surface coverage of the thrombi was analyzed by calculating the area covered by three-dimensional thrombi using the Carl Zeiss Software “ZEN 2012“ (blue edition).

### 4.8. Total Thrombus-Formation Analysis System (T-TAS^®^)

The T-TAS^®^ (Fujimori Kogyo Co. Ltd., Tokio, Japan) is a microchip-based whole blood flow chamber system to analyze in vitro thrombus formation [42,43,44]. Whole blood collected from mice supplemented with Ca-Corn Trypsin Inhibitor (Fujimori Kogyo Co. Ltd., Tokio, Japan) was perfused over a microchip (AR-Chip) (Fujimori Kogyo Co. Ltd., Tokio, Japan) coated with collagen and thromboplastin at a flow rate of 240 s^−1^ and 600 s^−1^. An integrated pressure sensor continuously measured the pressure in the artificial vessel. In case of thrombus formation, the artificial vessel was occluded. At 100 kPa the experiment was ended owing to near complete occlusion of the artificial vessel due to thrombi, and the occlusion time (start of flow until 100 kPa was reached) was measured.

### 4.9. Histology and Fluorescence Microscopy

After FeCl_3_-induced injury, the occluded carotid artery was removed and embedded in paraffin. Paraffin sections were stained with biglycan-specific antibody (biglycan polyclonal rabbit antibody, abcam, Cambridge, United Kingdom, # 49701, 1:100) following the streptavidin–biotin immuno-peroxidase method (Dako, Glostrup, Denmark). Sample slices of healthy carotid arteries were prepared from paraffin-embedded samples, dewaxed and dehydrated, blocked (5% goat serum, 0.1% BSA and 0.3% triton (Sigma-Aldrich, Burlington, MA, USA, # T8787-100)), and incubated with antibodies against collagen I (rabbit anti-mouse collagen I antibody, Thermo Fisher Scientific Inc., Waltham, MA, USA, # PA1-85319) and collagen IV (rabbit polyclonal collagen IV antibody, abcam, Cambridge, United Kingdom, # ab6586). Then, secondary antibody incubation was performed for 1 h (Alexa Fluor™ 555 goat anti-rabbit IgG (Thermo Fisher Scientific Inc., Waltham, MA, USA # A21428)), and cell nuclei were stained with DAPI (Roche Diagnostics, Mannheim, Germany, final concentration 1.6 µg/mL) and inundated with FluoromountTM (Sigma-Aldrich, Burlington, MA, USA). The documentation was done after drying at 4 °C overnight with microscope Axio Observer.D1 (Carl Zeiss Microscopy GmbH, Oberkochem, Germany, AxioCam MRm, objective LD Plan-Neofluar 40× 0.6 Korr. Ph2 M27).

### 4.10. Biglycan ELISA

Biglycan levels in the supernatant of stimulated platelets were determined using an ELISA (Cusabio Technology LLC, Houstomn, TX, USA # CSB-EL002683MO) according to the manufacturer’s protocol. Blood from C57BL/6J mice was collected in 200 μL acid-citrate-dextrose buffer (85 mM Na_3_-Citrat, 71 mM citric acid, 2% glucose (all purchased from Carl Roth GmbH + Co.), pH 4.69), and platelets were isolated as described above. Platelets (7 × 10^5^) were stimulated with CRP-XL (University of Cambridge, United Kingdom) or thrombin (Roche, Basel, Switzerland, # 10602400001) for 10 min at 37 °C, followed by centrifugation at 845× *g* for 10 min at 4 °C. Murine Tyrode’s buffer supplemented with CaCl_2_ (0.1 M) was added to the control group. The obtained supernatant was used for the ELISA.

### 4.11. RT-PCR

Isolation of RNA from murine platelets (C57BL/6J) was performed as described in 4.2. A minimum of 1 billion platelets were pooled from at least 3 mice for each biological replicate, while the purity of the platelet isolation was checked using a Sysmex cell analyzer. Subsequently, platelets were lysed using 500 µL TRI Reagent^®^ (Sigma-Aldrich, Burlington, MA, USA, #T9424) incubated for 5 min at RT. Chloroform (100 µL) was added, samples were vortexed and incubated for 3 min at RT following centrifugation at 12,000× *g* at 4 °C. The upper phase was collected and mixed with 250 µL isopropanol and incubated for 10 min at RT following a centrifugation at 12,000× *g* at 4 °C. Afterwards, supernatant was discarded and the pellet was washed twice with 75% ethanol following centrifugation at 7500× *g* for 5 min. Finally, the pellet was dried for 5–10 min at RT and dissolved in 40 µL RNAse-free water. RNA concentration was measured with an Eppendorf BioPhotometer^®^ D30 using 2 µL sample in an Eppendorf µCuvette^®^ G1.0. Purity of the RNA was checked with the 260/230 nm and 260/280 nm ratio. Afterwards, samples were stored at −80 °C until use.

RNA isolation from murine hearts of wildtype and knockout mice was performed with the ReliaPrepTM RNA Tissue Miniprep System (Promega, Walldorf, Germany, # Z6111) according to the manufacturer’s protocol. Complementary DNA was synthesized by using the ImProm- II™ Reverse Transcription System Kit (Promega, Walldorf, Germany, # A3800) following the manufacturer’s protocol. The qRT-PCR was performed by using the 7500 Fast Real-Time PCR System (Applied Biosystems by Thermo Fisher Scientific, Waltham, MA, USA). SYBR Green (Fast SYBRTM Green Master Mix, Applied Biosystems by Thermo Fisher Scientific Inc., Waltham, MA, USA, # 4385162) was used as a fluorescence dye. Actin was used as housekeeping gene. Quantitative PCR amplification was performed using the following oligonucleotide primers: biglycan for “ctgagggaacttcacttgga” rev “cagatagacaacctggaggag”; actin for “ctaaggccaaccgtgaaaag” rev “accagaggcatacagggaca”.

### 4.12. Calibrated Automated Thrombography

The endogenous thrombin generation was determined using calibrated automated thrombography in murine platelet-poor plasma (PPP) as described previously by Tchaikovski et al. with slight modifications [45]. Briefly, murine blood was anticoagulated with sodium citrate (final concentration 0.32%), followed by two times centrifugation at 21,000× *g* for 10 min at 22 °C to obtain PPP. PPP-reagent LOW, thrombin calibrator, and FluCa-Kit were purchased from Stago Deutschland GmbH (Düsseldorf, Germany). PBS was used to dilute PPP-reagent LOW and thrombin calibrator each at a ratio of 10/15 with PBS. In the experiment, 25 μL of diluted PPP-reagent LOW or thrombin calibrator was mixed with 15 μL PPP and incubated for 10 min at 33 °C. Subsequently the reaction was started by adding 20 μL FluCa buffer. The measurement of the fluorescent signal in triplicates at 33 °C for 30 min was made with a Fluoroscan Ascent plate reader (excitation: 390 nm, emission: 460 nm; Thermo Labsystems), and automatic data analysis was performed with Thrombinoscope™ Software (Stago Deutschland GmbH, Düsseldorf, Germany).

### 4.13. Western Blot Analysis

Western blot analysis was performed as described earlier [40] to detect GPVI-dependent phosphorylation in platelets upon incubation with recombinant human biglycan (R&D Systems, 2667-CM-050), and biglycan plus CRP. Platelets were separated by centrifugation, and lysates were made, separated on a gradient SDS polyacrylamide gel, and transferred onto nitrocellulose membrane. The membrane was blocked using 5% powdered skim milk in PBST (PBS with 0.1% Tween 20) and probed with anti-phosphotyrosine antibody, clone 4G10^®^ (Merck KgaA, Darmstadt, Germany, 05-321). The Novex Wedge Well kit (Thermo Fisher Scientific Inc., Waltham, MA, USA) was used as described in the manufacturers’ protocol.

### 4.14. Generation of Bone Marrow Chimera

The bone marrow donors (*Bgn^-/0^* and wildtype mice) were sacrificed by cervical dislocation, and the bone marrow cells were isolated from the tibias and femurs. After centrifugation at 500× *g* for 10 min, the remaining pellet was resuspended in red blood cell lysis buffer (155 mM NH_4_Cl (Merck KgaA, Darmstadt, Germany), 10 mM KHCO_3_ (Sigma-Aldrich, Burlington, MA, USA), 0.1 mM EDTA (Sigma-Aldrich, Burlington, MA, USA), pH 7.2–7.4, adjusted with HCl (Merck KgaA, Darmstadt, Germany)), followed by centrifugation at 500× *g* for 10 min. The pellet was resuspended in 500 μL PBS (Sigma-Aldrich Burlington, MA, USA). The recipients of bone marrow cells (12-week-old *Bgn^-/0^* mice and wildtype littermates) were preconditioned by irradiation with 10 gray (Gy) for 3.25 min using the Biobeam GM 2000 (Gamma-Service Medical GmbH, Leipzig, Germany). Irradiated *Bgn^-/0^* mice received isolated bone marrow cells from wildtype littermates, and irradiated wildtype littermates received isolated bone marrow cells from *Bgn^-/0^* mice (each 5 × 10^6^ cells). The animals were treated with neomycin (65.2 mg/50 mL drinking water) for 2 weeks to reduce the risk of infections. Six weeks after transplantation, the blood cells of the chimera were genotyped to confirm the successful generation of bone marrow chimeric mice. Moreover, an automated hematology cell counter (KX-21N) (Sysmex Deutschland GmbH, Norderstedt, Germany) was used to confirm the reconstitution of blood cells.

### 4.15. Genotyping of Blood Cells

The preparation of the eluates was performed with the ReliaPrepTM Blood gDNA Miniprep System (Promega, Walldorf, Germany, A5081). The KAPA Mouse Genotyping Kit (KAPA Biosystems Hoffmann-La Roche, Basel, Switzerland) was used according to the manufacturer’s protocol for the PCR. The generated pro-ducts (Bgn^-/0^, 319 bp; wildtype littermates, 212 bp) were detected with gel electrophoresis using a 1% agarose gel (Sigma-Aldrich, Burlington, MA, USA, A9539-250G)/TRIS-Acetate-EDTA (TAE)-buffer (50 × (121 g Trisbase (Sigma-Aldrich, Burlington, MA, USA), 28.5 mL acetic acid (100%, Merck KgaA, Darmstadt, Germany), 50 mL 0.5 M EDTA (Sigma-Aldrich, Burlington, MA, USA) pH = 8.3)) and Midori Green Advance (Nippon Genetics Europe GmbH, Düren, Germany, 617004).

### 4.16. Determination of Bleeding Time

Bleeding times in mice were determined as described in Gowert et al. (2017) [46]. Mice were anesthetized with Ketavet (90 mg/kg) (Zoetis Deutschland GmbH, Berlin, Germany) and Xylazin 2% (10 mg/kg) (Serumwerk Bernburg AG, Bernburg a. d. Saale, Germany) via intraperitoneal injection. The tip of the tail was amputated at a defined diameter (1.5 mm) using a gauge. The tail was immediately placed in a Falcon tube containing pre-warmed 0.9% NaCl, and bleeding was continuously monitored. The time was measured until bleeding had entirely stopped. In case of no cessation of bleeding and too much blood loss, the experiment was ended after 570 s and the bleeding stopped via cauterization.

### 4.17. Platelet Adhesion at the Injured Carotid Artery

This experiment was performed slightly modified as described in Grandoch et al. (2016) [13]. Platelets from Bgn^-/0^ mice and wildtype littermates were isolated. Platelets (8 × 10^6^) were stained with 5-Carboxyfluorescein diacetate N-succinimidyl ester (4.48 mM) (Sigma-Aldrich, Burlington, MA, USA, 87444-5MG-F). Bgn^-/0^ mice and wildtype littermates were anesthetized as described above. The right common carotid artery was dissected, the stained platelets injected into the retro-orbital plexus, and the wall of the carotid artery injured by performing a ligation with a surgical thread (PROLENE* Polypropylene Suture 6-0 (0.7 Ph. Eur., Ethicon, Inc., Raritan, NJ, USA, V396H) for 5 min. The ligation was re-opened, and the adhesion of the fluorescently-labeled platelets to the injured vessel wall was visualized at different time points by intravital microscopy (Leica Microsystems, Wetzlar, Germany).

### 4.18. Vessel Occlusion after FeCl_3_-Injury of the Carotid Artery

Wildtype and *Bgn^-/0^* mice and chimeras underwent FeCl_3_ injury of the carotid artery while anesthetized with ketamine (Zoetis) and xylacine (Serumwerk Bernburg AG) and put on a heating pad. Arteria carotis communis dextra was prepared, placed into an ultrasonic flow probe (Transonic Systems, 0.5 PSB, AD Instruments, Sydney, Australia), and moisturized with 0.9% physiological saline while baseline blood flow was measured. A 0.5 × 1 mm filter paper (Whatman No.1) saturated with 20% FeCl3 (Sigma-Aldrich, Burlington, MA, USA) was placed at the dried area of the carotid artery below the measuring head for 3 min, followed by recording of thrombus formation by the software (LabChart8, AD Instruments, Sydney, Australia) until 5 min after firm occlusion or until 30 min passed (in case of no occlusion). LabChart8Reader software (AD Instruments, Sydney, Australia) was used for analysis.

### 4.19. Expression of Surface Proteins

To analyze the expression of surface proteins, 100 μL murine blood was collected in 300 μL heparin (20 U/mL) (Ratiopharm GmbH, Ulm, Germany # 002304). An amount of 300 μL murine Tyrode’s buffer was added. For each approach, 30 μL of the diluted blood with 5 μL of FITC-labeled rat anti-mouse GPVI monoclonal antibody (M011-1), Phycoerythrin (PE)-labeled rat anti-mouse GPIbα (M040-2), FITC-labeled rat anti-mouse/human integrin β_3_ monoclonal antibody (M031-1) or FITC-labeled rat anti-mouse/human integrin α_5_ chain monoclonal antibody (M080-1) (all Emfret Analytics, Würzburg, Germany) were incubated for 15 min. Mean fluorescence intensity (MFI) was measured.

### 4.20. Clot Retraction Assay of Murine Platelet Rich Plasma

Clot retraction was performed with citrated murine PRP (300,000 platelets/μL) as described earlier [37]. Adding of thrombin (5 U/mL final; Roche, Basel, Switzerland, # 10602400001) and CaCl_2_ (20 mM final) to 250 μL of PRP started the clot formation. Reaction tubes were carefully mixed and incubated at 37 °C. Pictures were taken at indicated time points. Retraction was quantified by measuring the remaining fluid in relation to the starting volume.

### 4.21. Statistical Analysis

All statistical tests were performed using Graph Pad Prism software 7.0 (Graphpad Holdings LLC, San Diego, CA, USA) following its recommendations based on experimental design. Data are demonstrated as mean ± standard error of the mean (SEM). Statistical significance was analyzed by Student´s two-tailed, unpaired and paired *t*-tests, one-way or two-way ANOVA followed by the recommended post hoc test, in case of normality and otherwise by two-tailed Wilcoxon matched-pairs signed-rank test or for unpaired samples Mann–Whitney test. For the survival curve comparison in carotid artery, occlusion log-rank (Mantel–Cox) test was applied. *p*-values < 0.05 were considered to be statistically significant.

## Data Availability

The datasets generated during and/or analysed during the current study are available from the corresponding author on reasonable request.

## Supplementary Materials

The following are available online at https://www.mdpi.com/article/10.3390/ijms222212168/s1.

## Abbreviations

ADP	Adenosine 5´-diphosphate
ApoE	Apolipoprotein E
BGN	Biglycan
BSA	Bovine serum albumin
CD	Cluster of differentiation
Coll.	Collagen
CRP	Collagen related peptide
ECM	Extracellular matrix
GTP	Guanosine triphosphate
GP	Glycoprotein
MFI	Mean fluorescence intensity
PBS	Phosphate-buffered saline
PLCγ2	Phospholipase Cγ2
PPP	Platelet poor plasma
PRP	Platelet rich plasma
TLR	Toll-like receptor
T-TAS^®^	Total Thrombus-formation Analysis System
VWF	Von Willebrand factor
WT	Wildtype
