# Supplementary material for: The Proteoglycan Biglycan Modulates Platelet Adhesion and Thrombus Formation in a GPVI-Dependent Manner
Source: Int J Mol Sci. 2021 Nov 10;22(22):12168. doi: 10.3390/ijms222212168 (PMC8622445; doi:10.3390/ijms222212168)
Supplement: Supplementary file 1 [file ijms-22-12168-s001.zip › ijms-1408856-supplementary.pdf]

International Journal of
Molecular Sciences

MDPI

Article

The proteoglycan biglycan modulates platelet adhesion and thrombus formation in a GPVI-dependent manner

Henrike Hoermann[1], Irena Krüger[1], Nadine Maurus[1], Friedrich Reusswig[1], Yi Sun[2], Christina Kohlmorgen[3], Maria Grandoch[3], Jens W. Fischer[3], Margitta Elvers[1*]

Supplemental Data

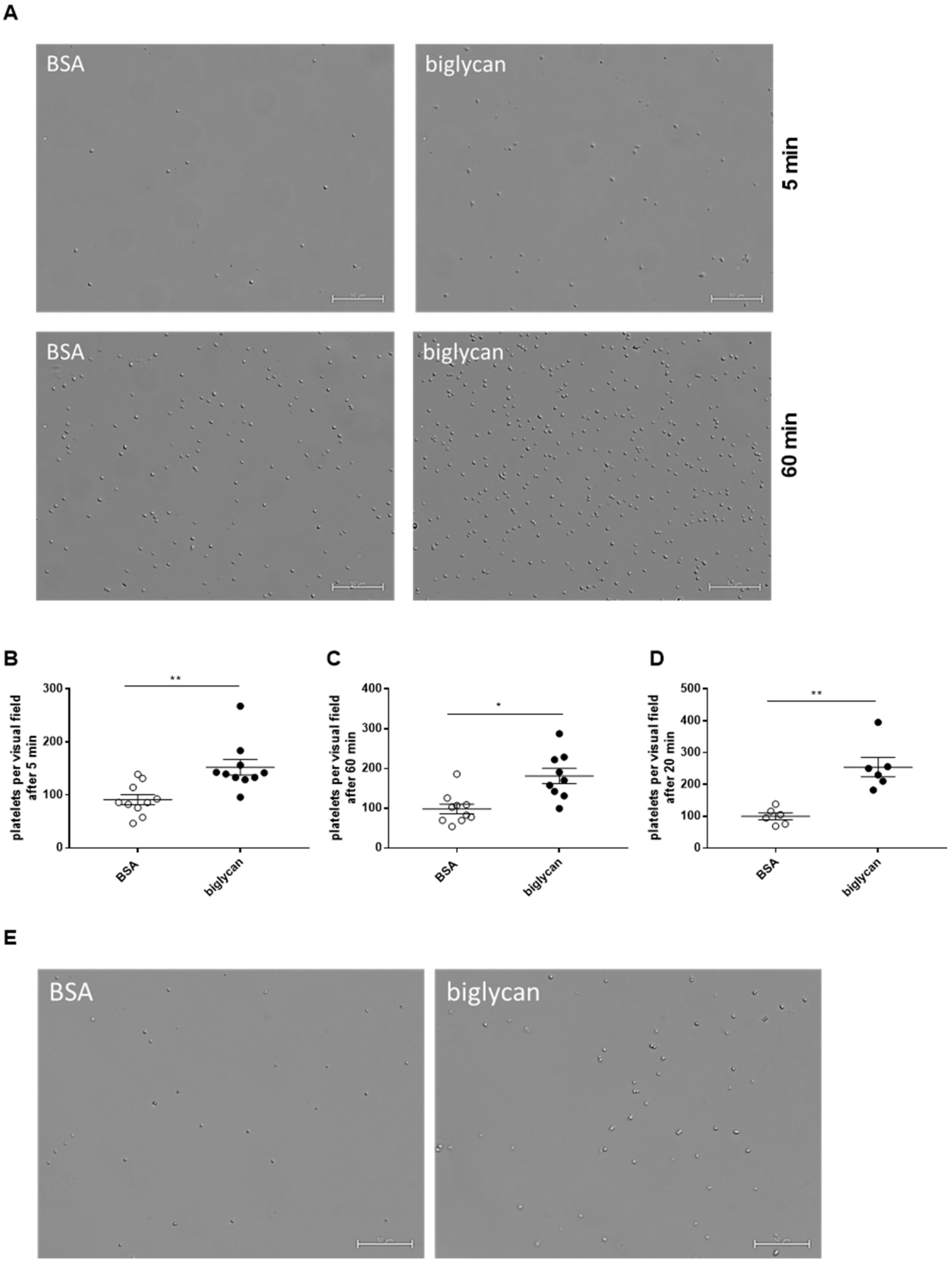

Suppl.-Figure S1. Specific adhesion of platelets to immobilized biglycan compared to BSA controls. (A-C) Adherent murine platelets after 5 and 60 min on BSA and biglycan. Representative pictures (A), quantitative analysis of adherent murine platelets on indicated matrices after 5 min (B) and 60 min (C). Data represent mean values ± SEM, n = 10, statistical analysis was performed with two-tailed Wilcoxon matched-pairs signed rank test. (D-E) Adhesion of human platelets on biglycan matrix compared to BSA control. (D) Quantification of adherent platelets on the indicated matrices after 20 min and (E) representative pictures, meanvalues ± SEM, n = 6. Statistical analysis was performed with two-tailed paired Student's t-test. Scale bar 50 μm. *P < 0.05, and **P < 0.01.

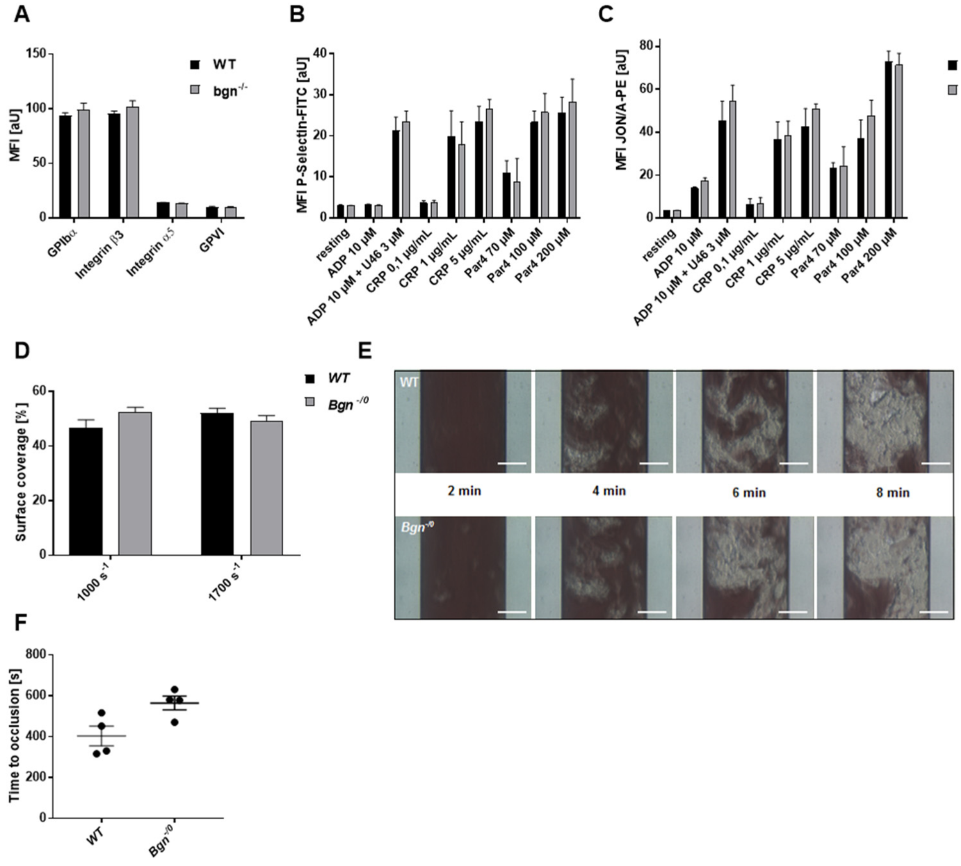

Suppl.-Figure S2. Unaltered platelet activation and thrombus formation in biglycan deficient mice. Glycoprotein expression (A), P-selectin exposure (B) and integrin $\alpha_{IIb}\beta_3$ activation (C) of platelets in washed whole blood of biglycan-deficient and WT mice. (D) Unaltered thrombus formation at shear rates of 1.000 s^{-1} and 1.700 s^{-1} using whole blood from *Bgn*$^{-/0}$ compared to WT mice *ex vivo*, bar graphs depict mean values ± SEM, n = 5-6. (E-F) Representative images show thrombus formation (E) and time to occlusion of the capillary in the T-TAS® system (F), scale bar = 100 μm.

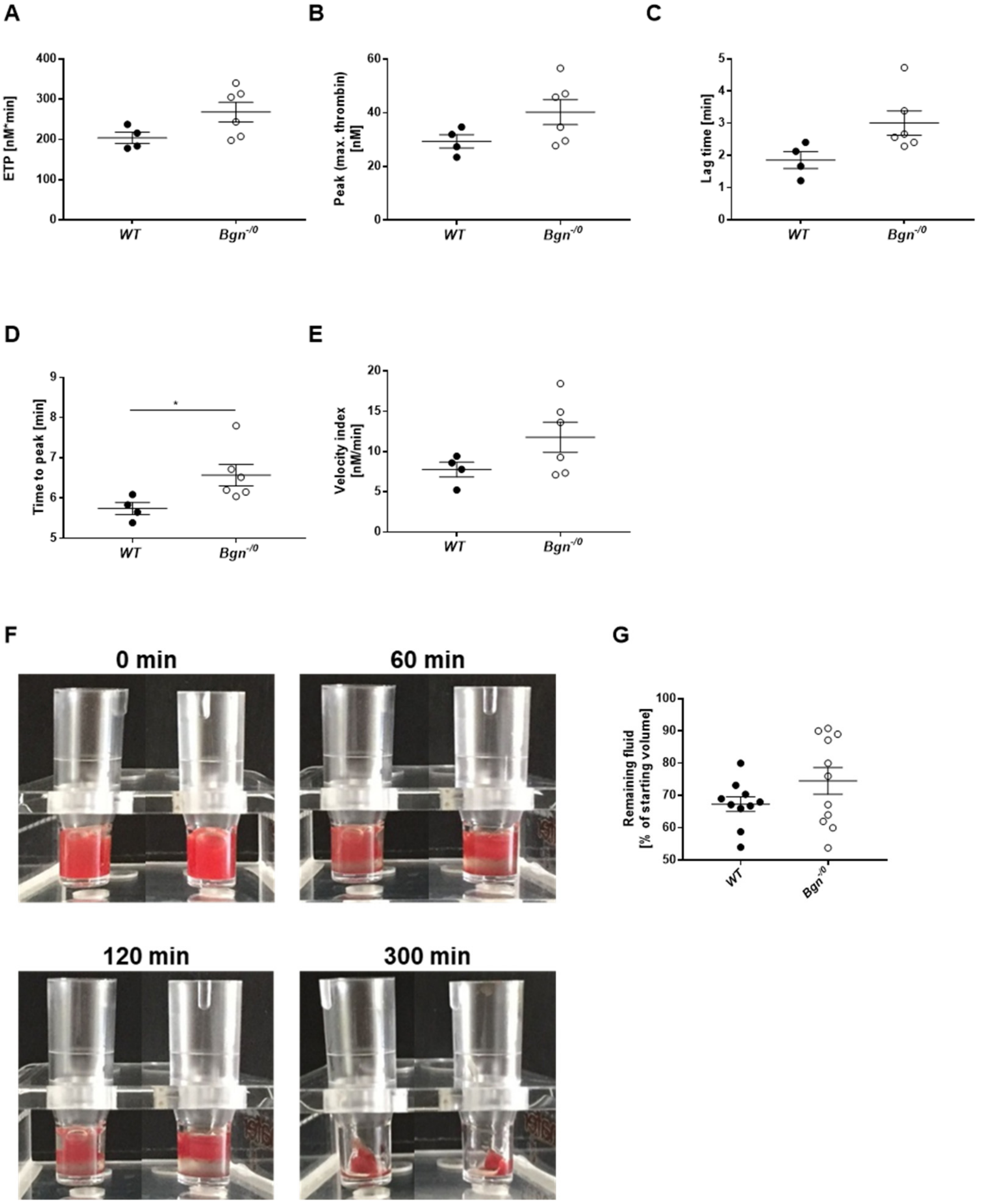

Suppl.-Figure S3. Unaltered thrombin plasma levels and normal clot retraction of platelets from *Bgn*$^{-/0}$ mice. (A) Endogenous thrombin potential (ETP) and (B) basal thrombin generation in PRP was measured with the fluorogenic calibrated automated thrombogram assay. (C) Lag time, (D) time to peak and (E) the velocity index are shown, data represent mean values ± SEM, n = 4-6. (F, G) Clot retraction was performed with platelet-rich-plasma (PRP) from *Bgn*$^{-/0}$ mice and wildtype littermates (WT). PRP was incubated with thrombin (5 U/mL) in the presence of $CaCl_2$ (20 mM). (F) Representative pictures of clot retraction were taken at indicated time points. (G) Retraction of the clots was quantified after 300 min by measuring the remaining fluid in relation to the starting volume, mean values ± SEM, n = 10-11.

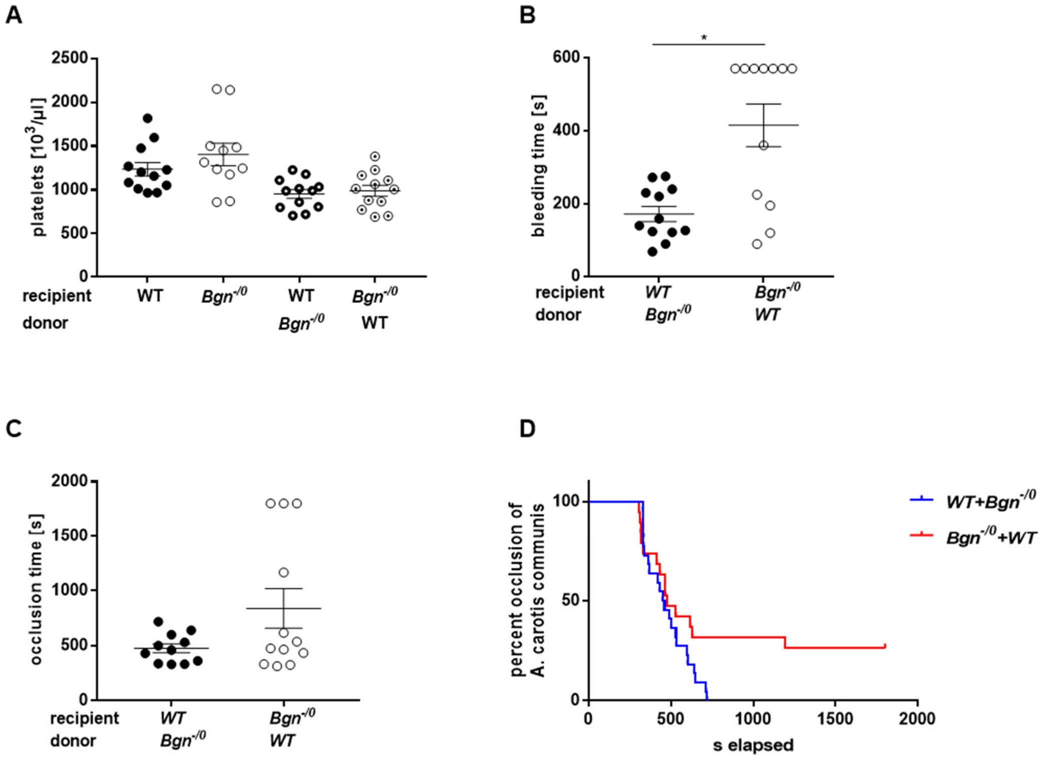

Suppl.-Figure S4. Altered hemostasis in bone marrow chimeras with biglycan-deficient mice that received bone marrow from wildtype controls. (A) Determination of platelet counts of bone marrow chimera compared to native WT and biglycan-deficient mice, mean values ± SEM, n = 11-12. (B) *Bgn*$^{-/0}$ mice that received bone marrow from wildtype controls showed enhanced bleeding times compared to WT mice that received bone marrow from biglycan-deficient mice. Time to cessation of bleeding is shown, data as mean values ± SEM, n = 12. Statistical analysis was perfomred with two-tailed Mann-Whitney test. (C-D) Time to occlusion of the carotid artery after $FeCl_3$-induced injury in bone marrow chimeras (C) and the percentage of vessel occlusion (D), mean values ± SEM, n=11-12. *P < 0.05.

Supplemental Table

	WT	*Bgn*$^{-/0}$
Platelet counts [x10^{6}/µL]	1.177 ± 102	1.186 ± 65
MPV	5.088 ± 0.044	5.138 ± 0.046
RBCs [x10^{6}/µL]	8.021 ± 0.439	7.937 ± 0.389
WBCs [x10^{3}/µL]	7.477 ± 1.342	7.095 ± 0.858

Suppl.-Table SI. Blood cell counts and MPV of wild-type and Bgn-/0 mice. Blood cell counts and MPV were determined with a hematology analyzer. There was no significant distinction in the shown parameters, mean values ± SEM, n = 8. MPV = mean platelet volume; RBC = red blood cell; WBC = white blood cell.
